# Supplementary material for: Carbohydrate-Responsive Element-Binding Protein-Associated Metabolic Changes in Chemically Induced Hepatocarcinogenesis Mouse Model
Source: Int J Mol Sci. 2025 Jul 18;26(14):6932. doi: 10.3390/ijms26146932 (PMC12295894; doi:10.3390/ijms26146932)
Supplement: Supplementary file 1 [file ijms-26-06932-s001.zip › ijms-3751827-supplementary.pdf]

Carbohydrate-responsive element-binding protein-associated metabolic changes in chemically induced hepatocarcinogenesis mice model

Maren Engeler et. al.

Supplementary Table S1: Percentage of glycogen-storing hepatocytes.

Glycogenosis (%)	4 weeks			12 weeks			36 weeks		
	n	mean	SEM	n	mean	SEM	n	mean	SEM
**KO DEN**	17	0	0	8	24,81 * §	9,75	22	6,03 * §	1,99
**L-KO DEN**	17	0	0	8	0 *	0	23	0 *	0
**WT DEN**	16	0	0	8	0 §	0	22	0 §	0
**KO Control**	20	8,62 * §	2,51	25	4,35 * §	1,25	24	3,29 * §	1,29
**L-KO Control**	25	0 *	0	25	0 *	0	25	0 *	0
**WT Control**	24	0 §	0	24	0 §	0	25	0 §	0

* KO vs. L-KO; § KO vs. WT; and p < 0,05

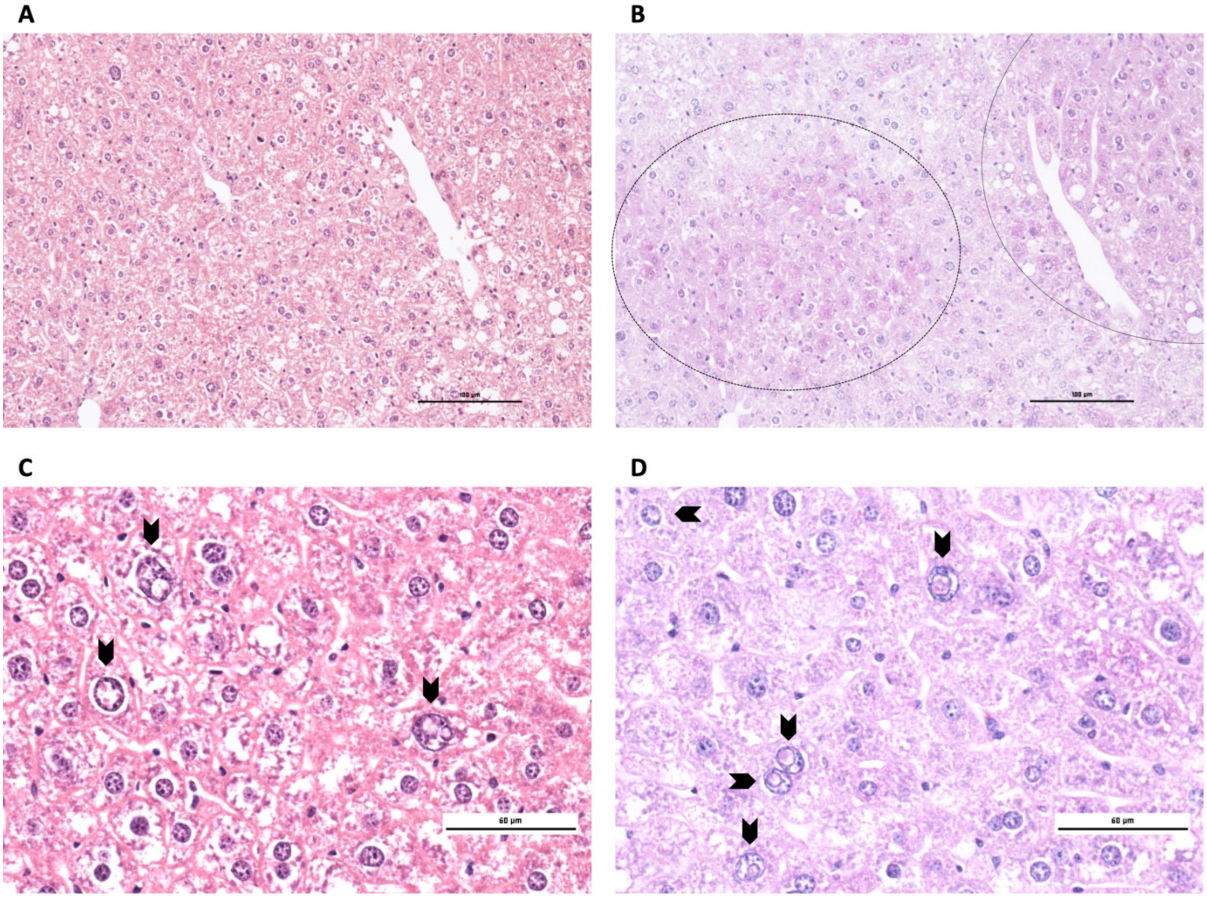

Supplementary Figure S1: Glycogen storage in unaltered hepatocytes. (A) An exemplary micrograph of an H&E-stained liver section of a ChREBP-KO DEN mouse after 4 weeks of testing with cytoplasmic glycogen storage. (B) The PAS reaction of the same section, ChREBP-KO DEN 4 weeks. The purple cytoplasm due to glycogen storage in hepatocytes. Images A-B are on the same scale, and the bar represents 100 µm. (C) An H&E-stained liver section with glycogen-storing cell nuclei (marked with arrows), (D) the same section in the PAS reaction. Images C-D are on the same scale, and the bar represents 60 µm.

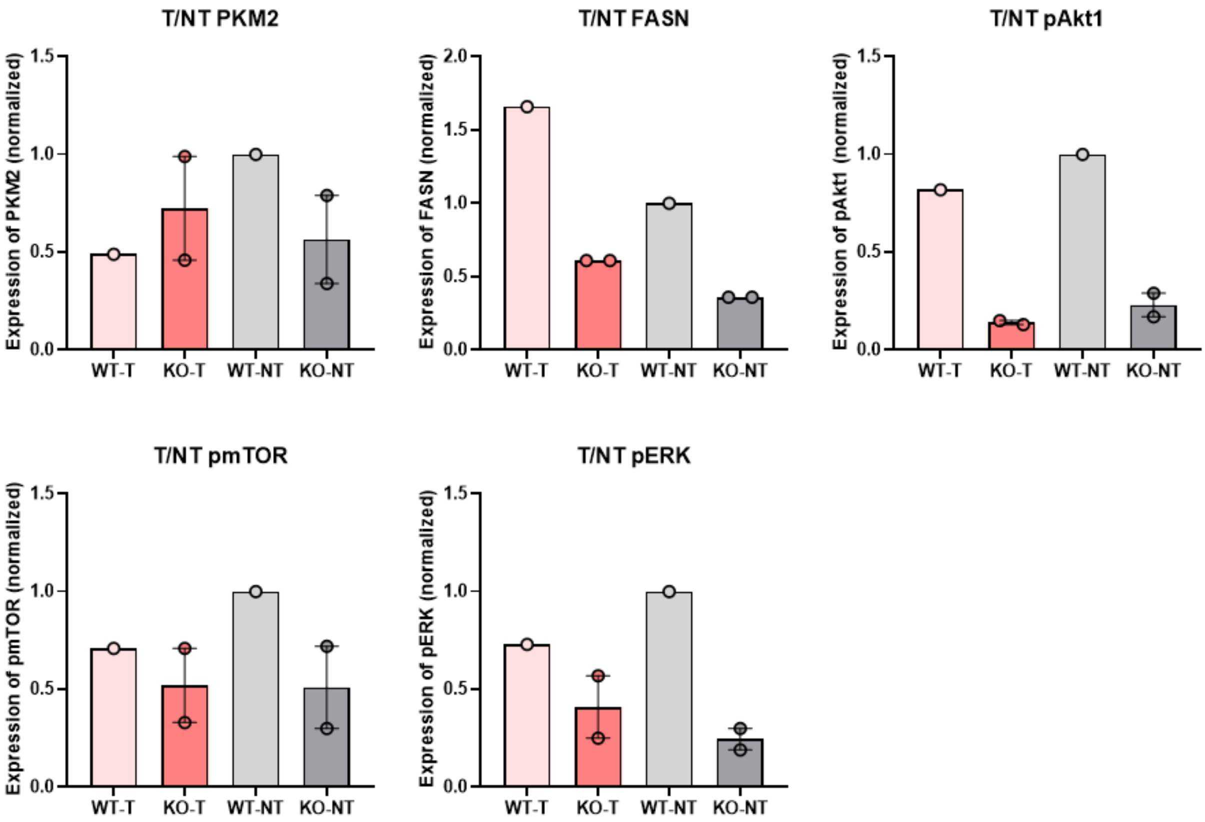

Supplementary Figure S2: Protein expression in tumors and unaltered liver tissue of wildtype and ChREBP-KO mice. The densitometric analysis of the Western blot presented in Figure 4 of the article. WT-T: Wildtype tumor after DEN application (n = 1); KO-T: ChREBP-KO tumor after DEN application (n = 2); WT-NT: Wildtype non-tumor tissue after DEN application (n = 1); and KO-NT: ChREBP-KO non-tumor tissue after DEN application (n = 2). Data are represented as the mean ± SEM. * $p < 0.05$. PKM2, pyruvate kinase M2; FASN, fatty acid synthase; pAkt1, phosphorylated protein kinase B coding gene; mTOR, mechanistic target of rapamycin; and pERK, phosphor-extracellular signal regulated kinase 1/2.

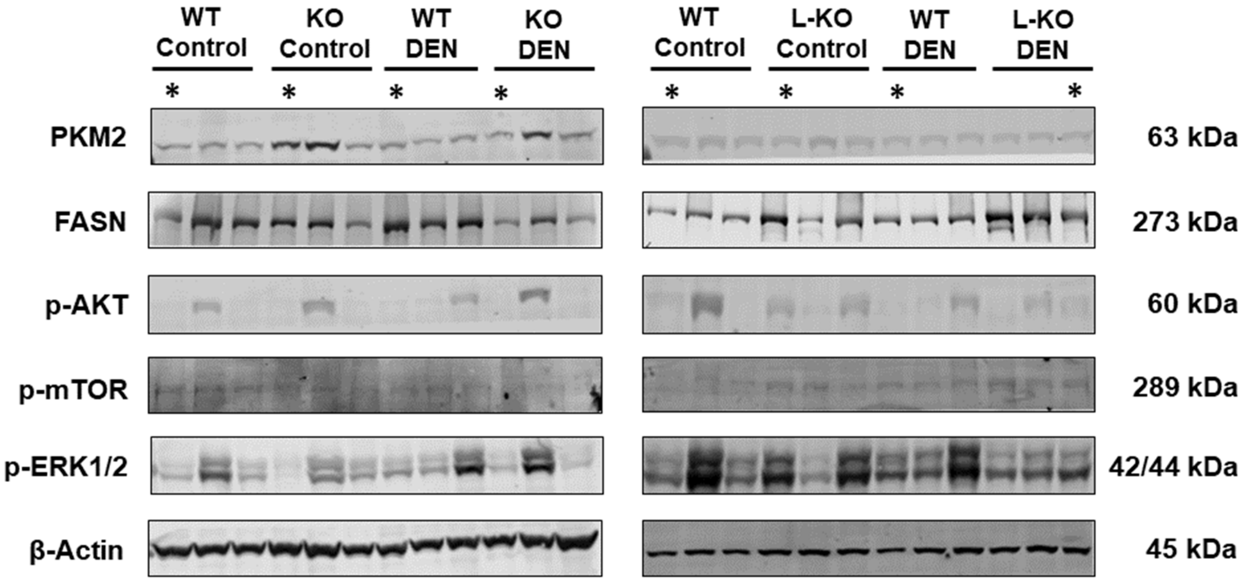

Supplementary Figure S3: The protein expression in the unaltered liver tissue of ChREBP-KO, Liver-ChREBP-KO, and WT mice of DEN and control groups after 36 weeks. Immunohistochemical stained mice marked with * are shown in Supplementary Figures 5 and 6. The p-mTOR blot on the left (WT and ChREBP-KO) is a rescan, as the quality of the first scan was too poor.

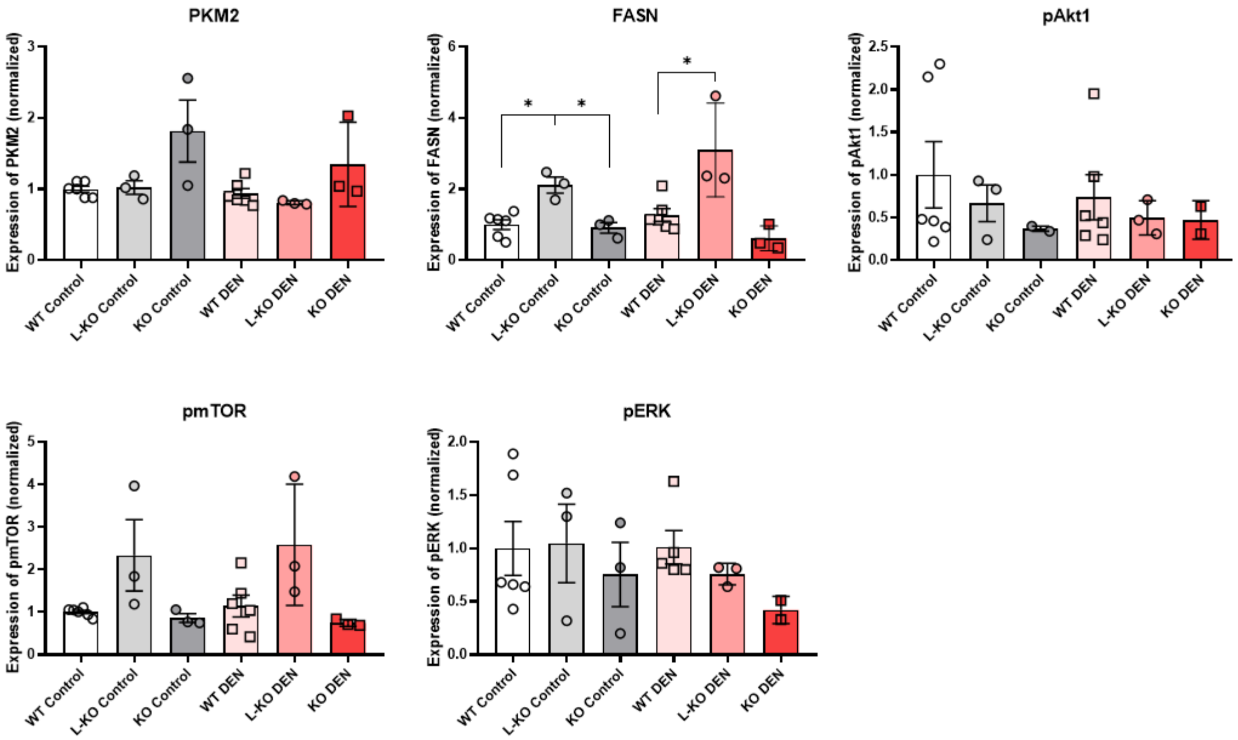

Supplementary Figure S4: The densitometric analysis of the Western blot presented in Supplementary Figure 3. The expression in unaltered liver tissue of ChREBP-KO, Liver-ChREBP-KO, and WT mice of DEN and control groups after 36 weeks (n = 6). Data are represented as the mean ± SEM. * $p < 0.05$. PKM2, pyruvate kinase M2; FASN, fatty acid synthase; pAkt1, phosphorylated protein kinase B coding gene; pmTOR, phosphorylated mechanistic target of rapamycin; and pERK, phosphor-extracellular signal regulated kinase 1/2.

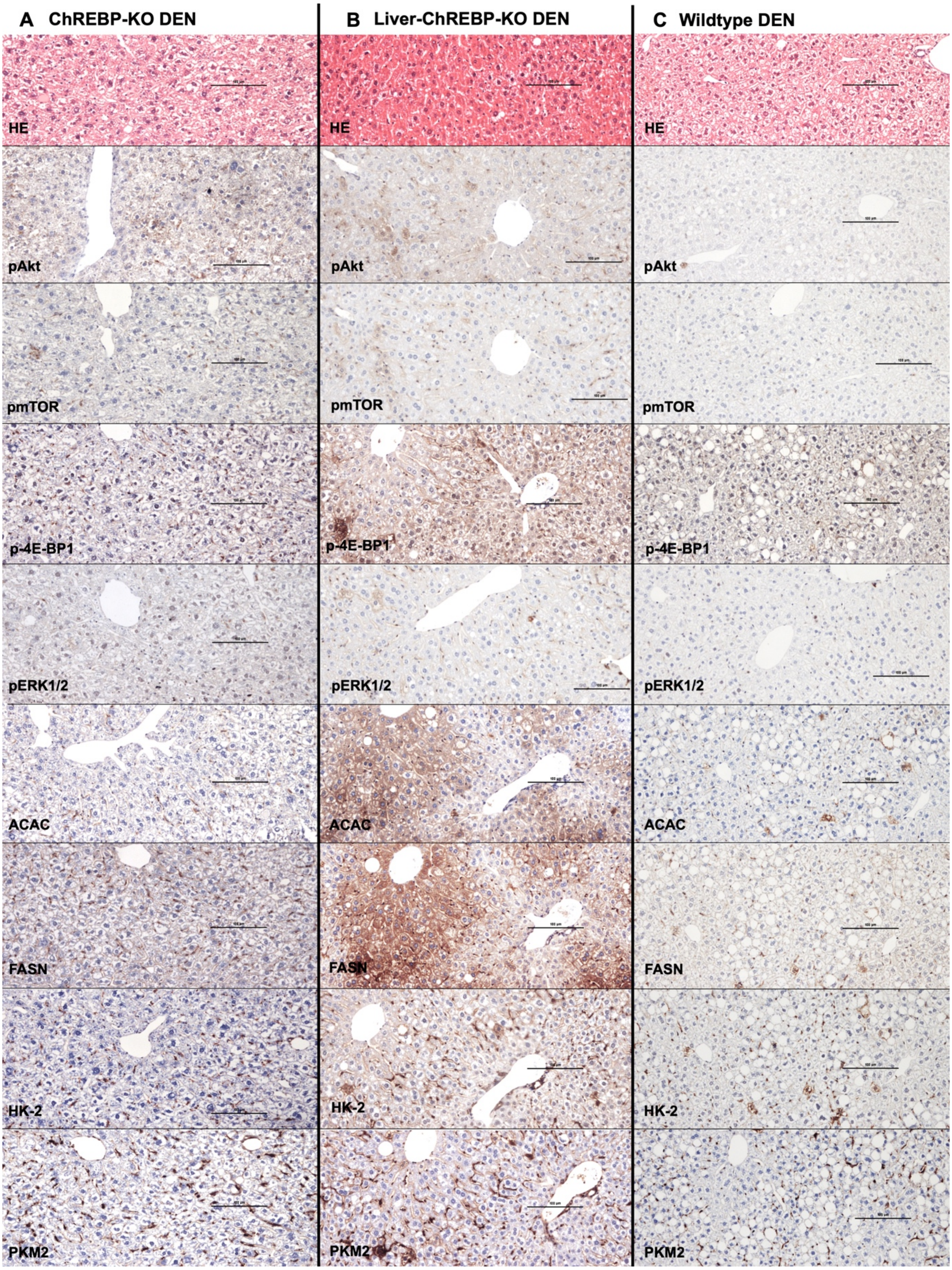

Supplementary Figure S5: Representative immunohistochemical findings in liver tissue of (A) ChREBP-KO, (B) Liver-ChREBP-KO, and (C) WT mice after DEN application and 36 weeks.

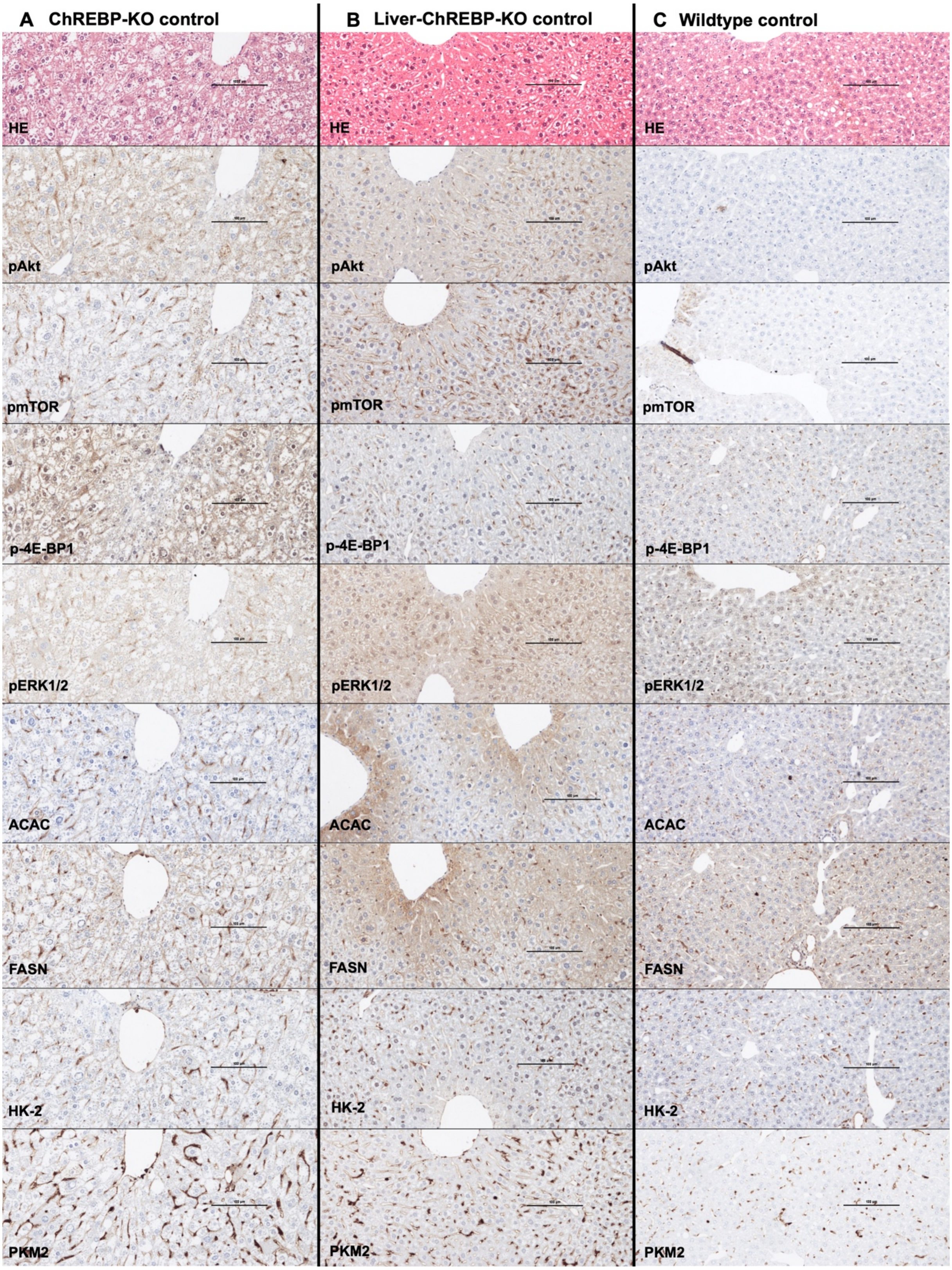

Supplementary Figure S6: Representative immunohistochemical findings in liver tissue of (A) ChREBP-KO, (B) Liver-ChREBP-KO, and (C) WT mice of the control groups and 36 weeks.

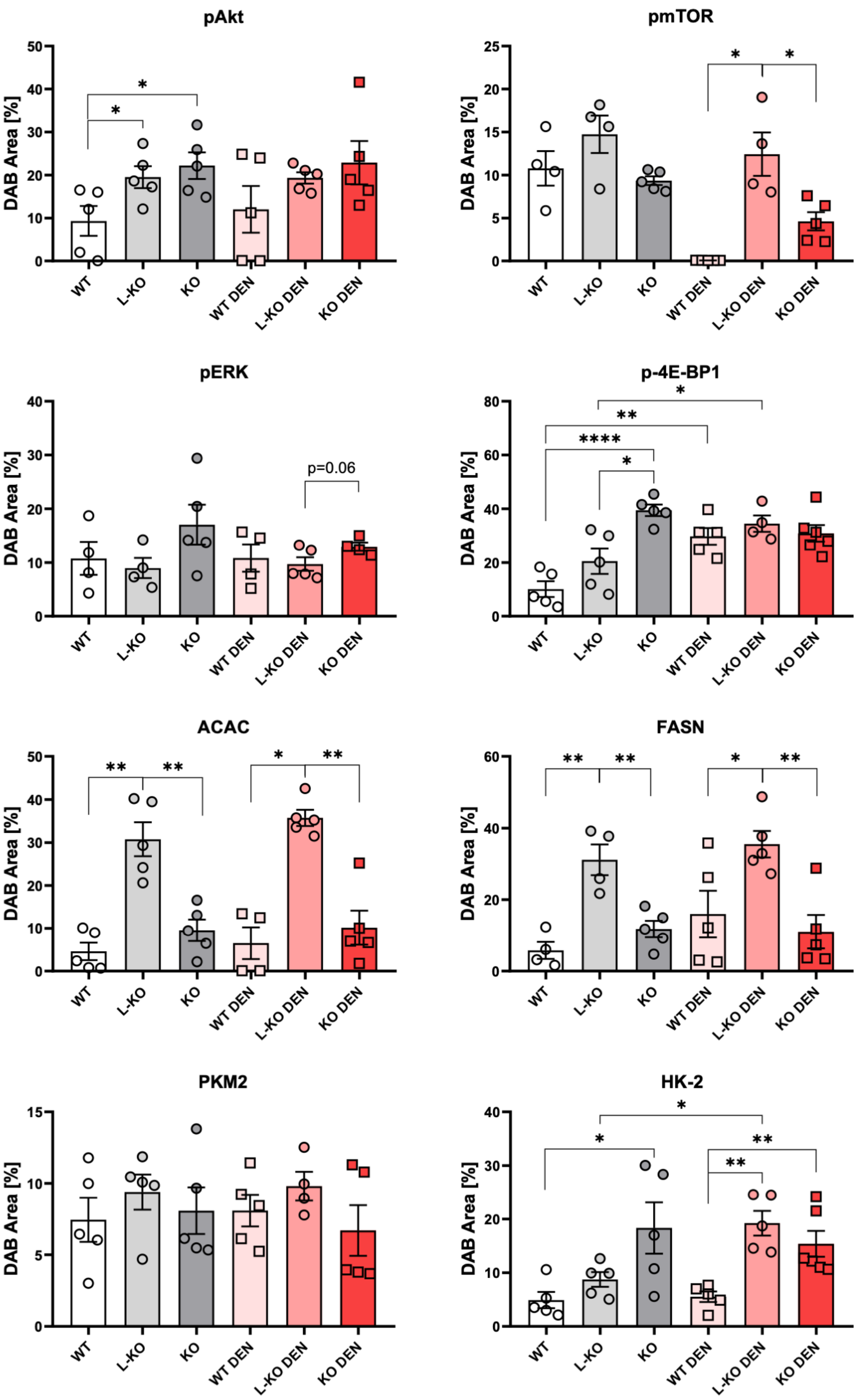

Supplementary Figure S7: Protein expression in immunohistochemically stained unaltered liver sections from each group (n = 5), quantified as stained area. Data are represented as mean ± SEM. * $p < 0.05$. pAkt, phosphorylated protein kinase B coding gene; pmTOR, phosphorylated mechanistic target of rapamycin; pErk, phosphor-extracellular signal regulated kinase; p-4E-BP1, phosphorylated eukaryotic translation initiation factor 4E binding protein 1; ACAC, acetyl-CoA carboxylase; FASN, fatty acid synthase; PKM2, pyruvate kinase M2; and HK-2, hexokinase II.

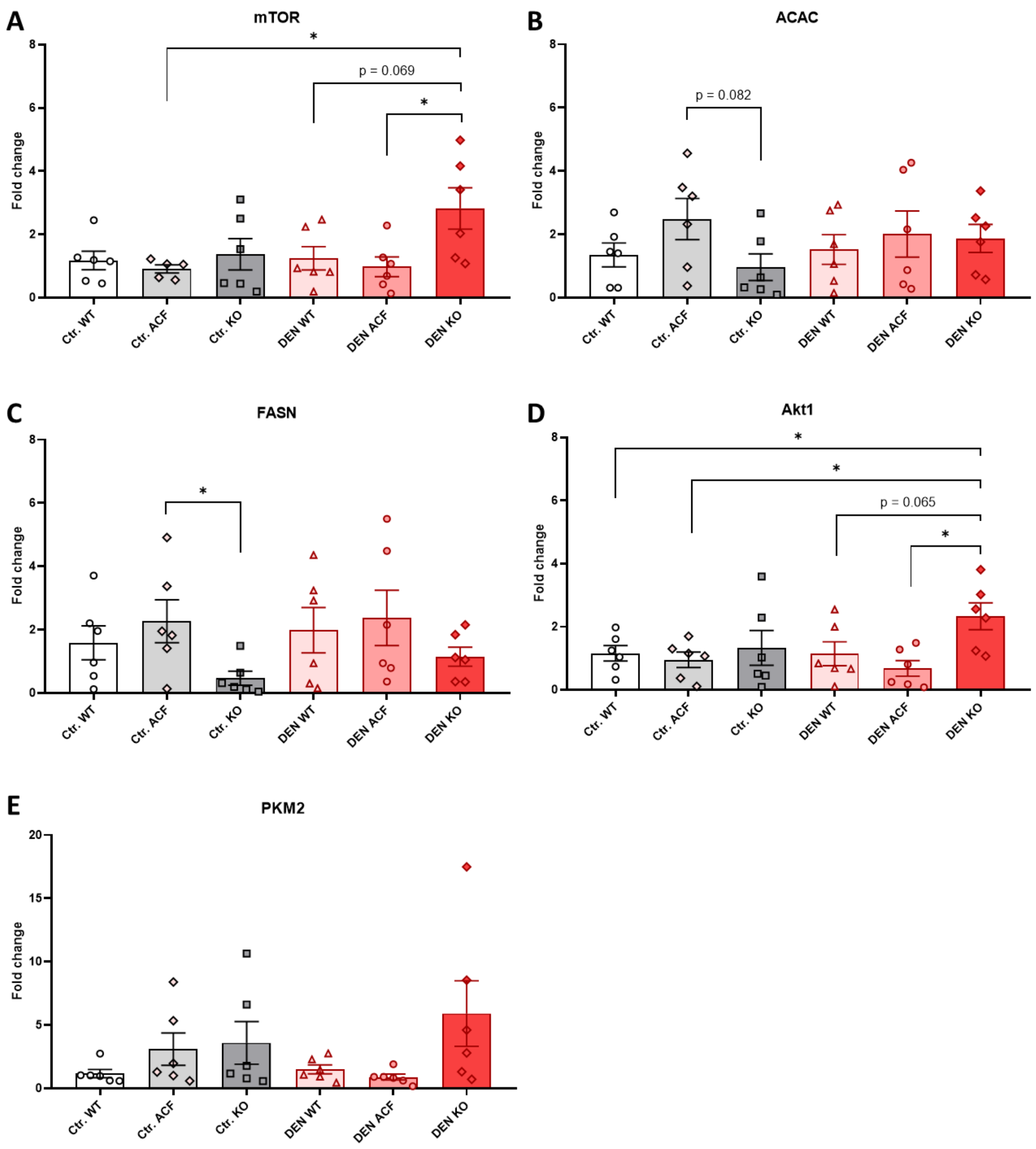

Supplementary Figure S8: Fold change gene expression in unaltered liver tissue determined by qPCR for each group (n = 6). Data are represented as mean ± SEM. * $p < 0.05$. mTOR, mechanistic target of rapamycin; Akt1, protein kinase B coding gene; ACAC, acetyl-CoA carboxylase; FASN, fatty acid synthase; and PKM2, pyruvate kinase M2.

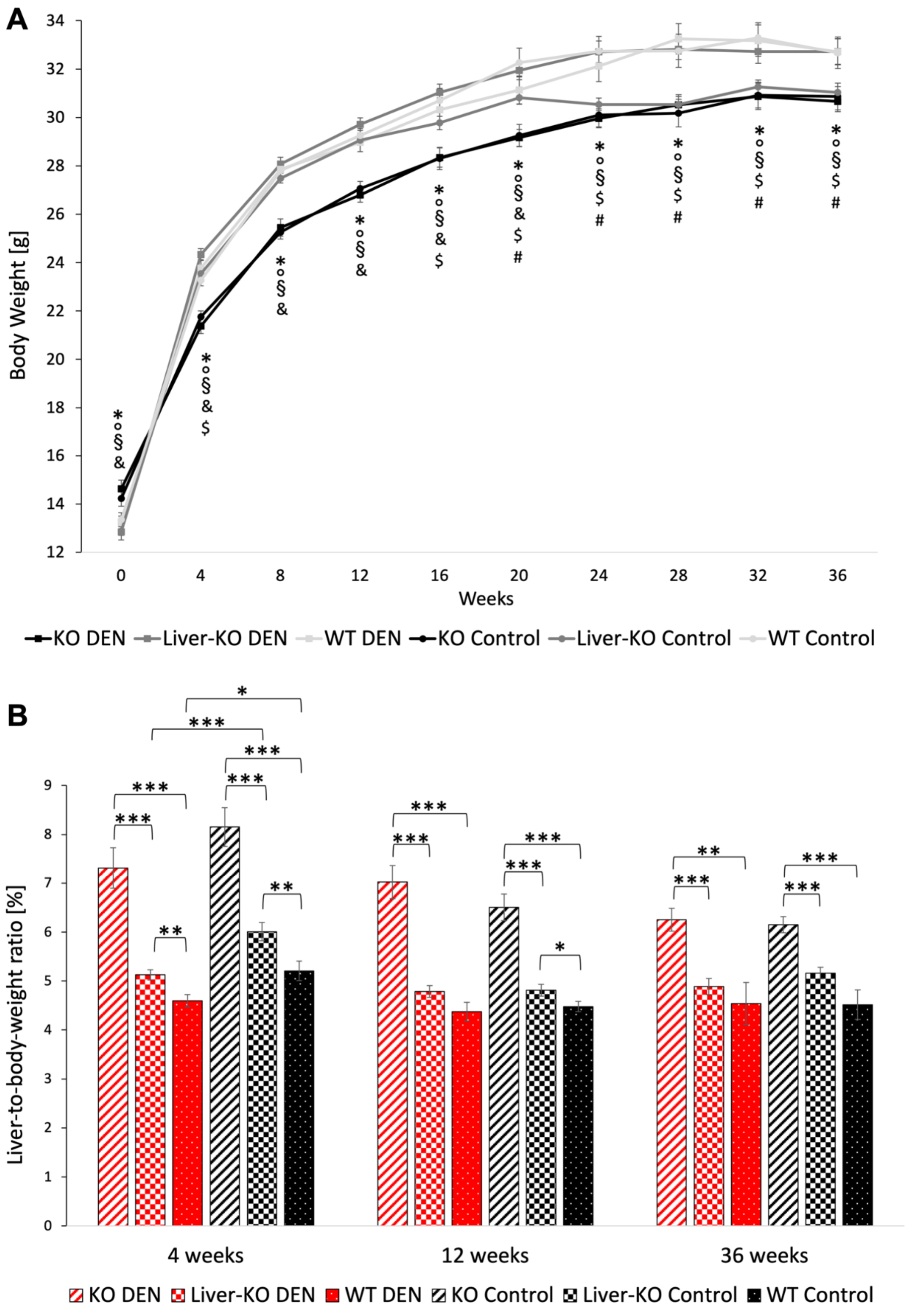

Supplementary Figure S9: Body weight progression and liver-to-body-weight ratio. (A) Body weight progression of ChREBP-KO, Liver-ChREBP-KO, and WT mice with DEN and control over 4, 12, and 36 weeks.* KO DEN vs. L-KO DEN , ° KO DEN vs. WT DEN, and KO control vs. L-KO control, § KO control vs. WT control, # L-KO control vs. WT control, $ L-KO DEN vs. L-KO control; $p < 0.05$ (B) liver-to-body-weight ratio of ChREBP-KO, Liver-ChREBP-KO, and wildtype mice with DEN and control after 4, 12, and 36 weeks. Data are represented as mean ± SEM. * $p < 0.05$; ** $p < 0.01$; and *** $p < 0.001$

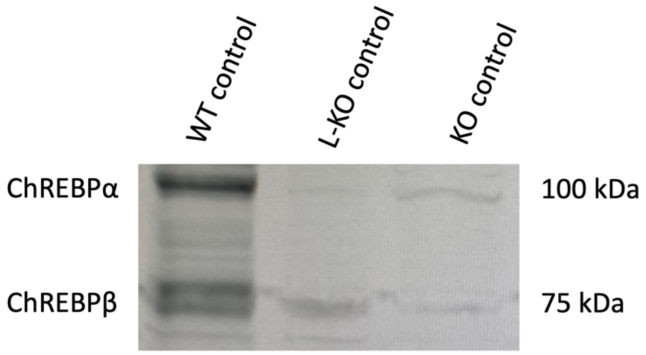

Supplementary Figure S10: The validation of the ChREBP-knockout. The Western blot analysis of ChREBP in liver whole-cell lysates of the wildtype control, liver-ChREBP-knockout control, and ChREBP-knockout control mice.

Supplementary Table S2: Diet composition.

	**V1534-000**
Fat, kcal%	9.0
Protein, kcal%	24.0
Carbohydrate, kcal%	67.0
Metabolizable Energy, MJ/kg	13.5
Crude Nutrients	
Crude protein, %	19.0
Crude fat, %	3.3
Crude fiber, %	4.9
Crude ash, %	6.4
Starch, %	35.9
Sugar, %	5.4

producer: ssniff Spezialdiäten, Soest, Germany

Supplementary Table S3: Primer information for genotyping.

**Primer name**	**Primer Sequence (5'-3')**
A (11742*)	GAC CAA CAC CCA ACA CCA G
AB_2	CCC CTG AGG ACT TTT GCT GT
B (oIMR4216*)	CGC CTT CTT GAC GAG TTC
AB (11421*)	CGG AGC CAC GCC TCT AA
Chrebpgeno2-31	CAA GCA AGT GAT GGG ACA CA
Chrebpgeno2-51	ACC CAC CTC TTC GAG TGC T
AlbCre R	TTG GCC CCT TAC CAT AAC TG
AlbCre Cre F	GAA GCA GAA GCT TAG GAA GAT GG
AlbCre WT F	TGC AAA CAT CAC ATG CAC AC

* Original primer names according to jax.org

Supplementary Table S4: List of primary antibodies.

Protein	Antibody-ID	Host and Clonality	Dilution IHC	Dilution WB	Company
ACAC	3676	Rabbit, monoclonal	1:400		Cell Signaling
FASN	10624-2-AP	Rabbit, polyclonal	1:1000	1:1000	Proteintech
Hexokinase 2	22029-1-AP	Rabbit, polyclonal	1:4000		Proteintech
p-4E-BP1	2855	Rabbit, monoclonal	1:800		Cell Signaling
p-AKT	4060	Rabbit, monoclonal	1:100	1:1000	Cell Signaling
p-ERK1/2	4370	Rabbit, monoclonal	1:100	1:1000	Cell Signaling
PKM2	4053	Rabbit, monoclonal	1:400	1:1000	Cell Signaling
p-mTOR	2976	Rabbit, monoclonal	1:100	1:500	Cell Signaling
β-Actin	4970	Rabbit, monoclonal	-	1:2000	Cell Signaling
Ki-67	KI681C01	Rabbit, monoclonal	1:50	-	DCS

ACAC: acetyl-CoA carboxylase; FASN: fatty acid synthase; IRS-1: insulin receptor substrate 1; PKM2: pyruvatkinase M2; and p-mTOR: phosphorylated mechanistic target of rapamycin

Supplementary Table S5: Primer information for qPCR.

Primer name	Primer Sequence (5'-3')
18S	F: AGT CCC TGC CCT TTG TAC ACA<br>R: CGA TCC GAG GGC CTC ACT A
ACAC	F: ATG GGC GGA ATG GTC TCT TTC<br>R: TGG GGA CCT TGT CTT CAT CAT
AKT1	F: ATG AAC GAC GTA GCC ATT GTG<br>R: TTG TAG CCA ATA AAG GTG CCA T
FASN	F: CTG CCA CAA CTC TGA GGA CA<br>R: CGG ATC ACC TTC TTG AGA GC
mTOR	F: ACC GGC ACA CAT TTG AAG AAG<br>R: CTC GTT GAG GAT CAG CAA GG
PKM2	F: GCC GCC TGG ACA TTG ACT C<br>R: CCA TGA GAG AAA TTC AGC CGA G
